# Exploration of the Shared Gene and Molecular Mechanisms Between Endometriosis and Recurrent Pregnancy Loss

**DOI:** 10.3389/fvets.2022.867405

**Published:** 2022-05-06

**Authors:** Zhuang Ye, Qingxue Meng, Weiwen Zhang, Junli He, Huanyi Zhao, Chengwei Yu, Weizheng Liang, Xiushen Li, Hao Wang

**Affiliations:** ^1^Department of Rheumatology, The First Hospital of Jilin University, Changchun, China; ^2^Department of Pediatrics, Shenzhen University General Hospital, Shenzhen, China; ^3^Department of Obstetrics and Gynecology, Shenzhen University General Hospital, Shenzhen, China; ^4^Guangzhou University of Chinese Medicine, Guangzhou, China; ^5^School of Future Technology, University of Chinese Academy of Sciences, Beijing, China; ^6^Guangdong Key Laboratory for Biomedical Measurements and Ultrasound Imaging, School of Biomedical Engineering, Shenzhen University Health Science Center, Shenzhen, China; ^7^Shenzhen Key Laboratory, Shenzhen University General Hospital, Shenzhen, China

**Keywords:** endometriosis, recurrent pregnancy loss, WGCNA, PI3K-Akt signaling pathway, platelet activation, miRNAs-mRNAs

## Abstract

Endometriosis (EMs) is a common benign gynecological disease in women of childbearing age, which usually causes pelvic pain, secondary dysmenorrhea, and infertility. EMs has been linked to recurrent pregnancy loss (RPL) in epidemiological data. The relationship of both, however, remains unknown. The purpose of this study is to explore the underlying pathological mechanisms between EMs and RPL. We searched Gene Expression Omnibus (GEO) database to obtain omics data of EMs and RPL. Co-expression modules for EMs and RPL were investigated by using weighted gene co-expression network analysis (WGCNA). The intersections of gene modules with the strong correlation to EMs or RPL obtained by WGCNA analysis were considered as shared genes. MicroRNAs (miRNAs) and their corresponding target genes linked to EMs and RPL were found though the Human MicroRNA Disease Database (HMDD) and the miRTarbase database. Finally, we constructed miRNAs-mRNAs regulatory networks associated with the two disorders by using the intersection of previously obtained target genes and shared genes. We discovered as significant modules for EMs and RPL, respectively, by WGCNA. The energy metabolism might be the common pathogenic mechanism of EMs and RPL, according to the findings of a Kyoto Encyclopedia of Genes and Genomes (KEGG) enrichment analysis. We discovered several target genes that might be linked to these two disorders, as well as the potential mechanisms. RAB8B, GNAQ, H2AFZ, SUGT1, and LEO1 could be therapeutic candidates for RPL and EMs. The PI3K-Akt signaling pathway and platelet activation were potentially involved in the mechanisms of EM-induced RPL. Our findings for the first time revealed the underlying pathological mechanisms of EM-induced RPL and identified several useful biomarkers and potential therapeutic targets.

## Introduction

Endometriosis (EMs) is a chronic, inflammatory, estrogen-dependent gynecological disease, the most typical symptom of which is the appearance of endometrial tissue in the non-uterine cavity (1). Retrograde menstruation, in which endometrial tissue flows back into the pelvis and subsequently scatters to various areas, producing the local inflammatory response and causing local tissue adhesions, is currently the most widely accepted pathologic explanation (2). Worldwide, approximately 8% of women of reproductive age suffer from EMs, which are accompanied by common clinical symptoms such as dysmenorrhea, pelvic pain, and infertility. Specific diagnostic markers, on the other hand, have yet to emerge, leading to delayed diagnosis of EMs (3). Although surgery is currently the gold standard for diagnosing EMs, patients should be aware of the risk of diminished ovarian reserve and recurrence of EMs as the results of surgical treatment. Oral medicine therapy, which is the most commonly recommended in the clinic, can only help with the symptoms of EMs. When pharmacological treatment is interrupted, the chances of recurrence are significant (4). Although EMs is a benign gynecological disease, they also have the risk of malignant transformation. In clinical practice, there is currently no satisfactory treatment approach.

The disorder having more than two miscarriages before 20–24 weeks of gestation is called recurrent pregnancy loss (RPL) (5). The lack of standardized early miscarriage diagnostic guidelines and the variable miscarriage symptoms of early miscarriage patients hinder the diagnosis and treatment of RPL. Patients with RPL have the better prognosis, but it is dependent on their age, mental health, and the number of losses (6). Even though aberrant uterine morphology, chromosomal structural abnormalities, and autoimmune disorders are all linked to the development of RPL, the underlying mechanisms of miscarriage in most RPL patients remain unknown (7). The therapy of RPL patients has been complicated by the lack of the recognized mechanisms of occurrence. EMs are associated with up to 50% of infertility patients and often lead to failure of assisted reproductive treatments (8). The mechanisms of EMs-induced RPL could be that inflammatory alterations in the endometrium of EMs patients impact predecidual transformation, eventually leading to placental abnormalities (9). However, the mechanisms through which EMs cause RPL are far from flawless.

With the widespread adoption of high-throughput transcriptome sequencing technology, we can now easily find differentially expressed genes in diseases, allowing us to gain the better understanding of disease onset and progression, as well as provide guidance for clinical disease treatment. In this study, We used different bioinformatic analysis approaches, including weighted gene co-expression network analysis (WGCNA), to analyze datasets linked to EMs and RPLs in the Gene Expression Omnibus (GEO) database. Finally, we found that the underlying mechanisms of EM-induced RPL were discovered to be connected to the PI3K/AKT signaling pathway and platelet activation.

## Methods

### Research Process

**Figure 1:**
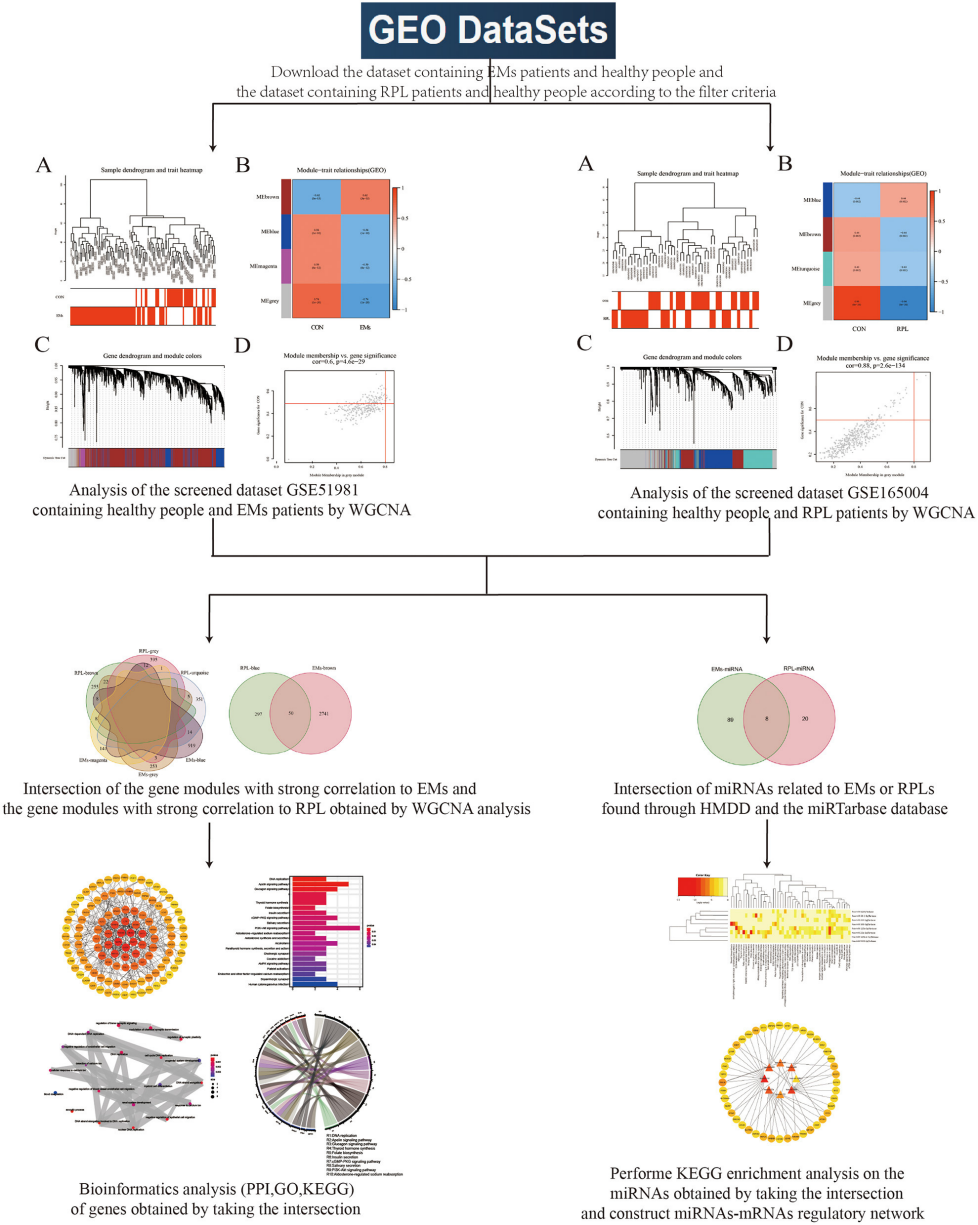
Flowchart. This figure depicted the process of using bioinformatics methods such as WGCNA analysis to look for shared genes and biological processes between EMs and RPL.

Figure 1 depicted the flowchart of this study. Transcriptome sequencing data for EMs and RPL were obtained from the GEO database. WGCNA was used to investigate gene modules linked to EMs and RPL, respectively. Shared genes were defined as intersection of gene modules with high correlation to EMs and RPL obtained by WGCNA. Find the related microRNAs (miRNAs) of EMs and RPL. Shared genes and miRNAs were subjected to bioinformatics analysis. Finally, genes and molecular mechanisms linked to EMs and RPL had been discovered.

### Screening of the GEO Dataset

We searched the GEO database with the keywords “endometriosis” and “recurrent pregnancy loss” respectively. The screening conditions of the dataset were as follows: (1) mRNA mRNA sequencing data from healthy people and diseased (EMs or RPL) patients, each with a population of more than 10, must be included in datasets; (2) The tissue used for sequencing should be the endometrium; (3) The sample's expression matrix file should be included in the dataset for further analysis.

### WGCNA

WGCNA, which uses phenotypic weight parameters, scale-free clustering, and dynamic shear trees to evaluate data, is frequently employed to investigate disease-related gene modules. The GSE51981 and GSE165004 datasets, respectively, contained transcriptome sequencing data for about 20,000 and 30,000 genes. Since the number of genes in the datasets was different, we took the intersection of the genes in the two datasets. Most of the genes in the GEO datasets had no difference in expression among the healthy and disease groups, so we selected the top 5,000 genes with significant differences for subsequent WGCNA analysis according to the *p*-value calculated by the R language “limma” package. We used the R language “WGCNA” package to uncover gene modules linked to EMs and RPL in this study. The “Hclust” function in the R language was used to eliminate outliers in the GSE51981 and GSE165004 datasets before starting the WGCNA analysis. Then, the “pickSoftThreshold” function in the R language was used to filter the soft threshold from 1 to 30. By modifying the adjacency matrix according to the selected soft threshold, we obtained the topological overlap matrix (TOM) and the corresponding dissimilarity (1-TOM). Then, we set the minimum number of genes in the module to 50 and utilized the cut line (mergeCutHeight = 0.25) to merge comparable gene modules. Finally, we assessed the relationship between gene modules and disease.

### Identification the Shared Genes of EMs and RPL

We used absolute value of the correlation more than 0.4 as the screening threshold to obtain gene modules that were strongly associated with EMs and RPL, respectively. The gene modules positively and negatively correlated with EMs and RPL were intersected and visualized through the R language “Venn” package, respectively. We defined the genes obtained from the intersection as shared genes.

### Construction of Protein-Protein Interaction Network

As a main method in bioinformatics, protein-protein interaction (PPI) network can comprehensively analyze the relationship and function of multiple proteins in organisms. The STRING database could be used to study and predict numerous protein interactions. To investigate gene interactions, we entered shared genes into STRING data. The “Homo sapiens” species was chosen, and PPI analysis was carried out with the “Multiple proteins” analysis module. The PPI network was constructed with the minimum required interaction score greater than 0.15 as the screening condition. Then, We downloaded the protein interaction information of the PPI network and optimized it though Cytoscape software.

### GO Term and KEGG Pathway Enrichment Analysis

The Gene Ontology (GO) database is composed of three sub-databases: molecular function, biological process, and cellular composition, which are used to define and describe gene functions. Kyoto Encyclopedia of Genes and Genomes (KEGG) database is a comprehensive database including system information, genome information, and chemical information, among which the KEGG pathway database is the most commonly used bioinformatics database. Both GO term and KEGG pathway enrichment analysis was performed on shared genes by using the R language “cluster Profiler” package and visualized the results of the enrichment analysis by the R language “ggplot” package.

### Exploration of Shared miRNAs for EMs and RPL

As non-coding RNAs, miRNAs play the vital function in gene regulation. As a result, we searched for miRNAs linked to EMs and RPL to build a miRNAs-mRNAs regulatory network. Experimentally validated miRNAs associated with human diseases could be found in the Human MicroRNA Disease Database (HMDD). In this study, we took the intersection of EMs-related and RPL-related miRNAs from the HMDD database. The intersection of EM-related and RPL- related miRNAs were defined as shared miRNAs.

### Bioinformatics Analysis of Shared miRNAs

The miRtarbase database has been continuously updated as a repository for miRNAs-mRNAs targeting relationships since its inception in 2011. We searched the miRtarbase database for target genes of shared miRNAs and took intersections with shared genes. Then, the miRNAs-mRNAs regulatory network was constructed by Cytoscape software. The DIANA Tools website aggregates miRNAs and lncRNAs research data and provides a variety of non-coding RNA research tools. Therefore, we used the DIANA tools website's mirPath v3.0 program to conduct the KEGG pathway enrichment analysis of miRNAs.

## Results

### Information From the Filtered GEO Dataset

According to our screening criteria, datasets with the GEO numbers GSE51981 and GES165004 were obtained. We exclusively used the data of GSE51981 dataset, which contained 77 EMs patients and 34 healthy people. For the GSE165004 dataset, we only used the transcriptome sequencing data of 24 healthy people and 24 patients with RPL. The platform annotation file corresponding to the dataset was used to convert gene probes into gene names.

### WGCNA Results of EMs and RPL Datasets

**Figure 2:**
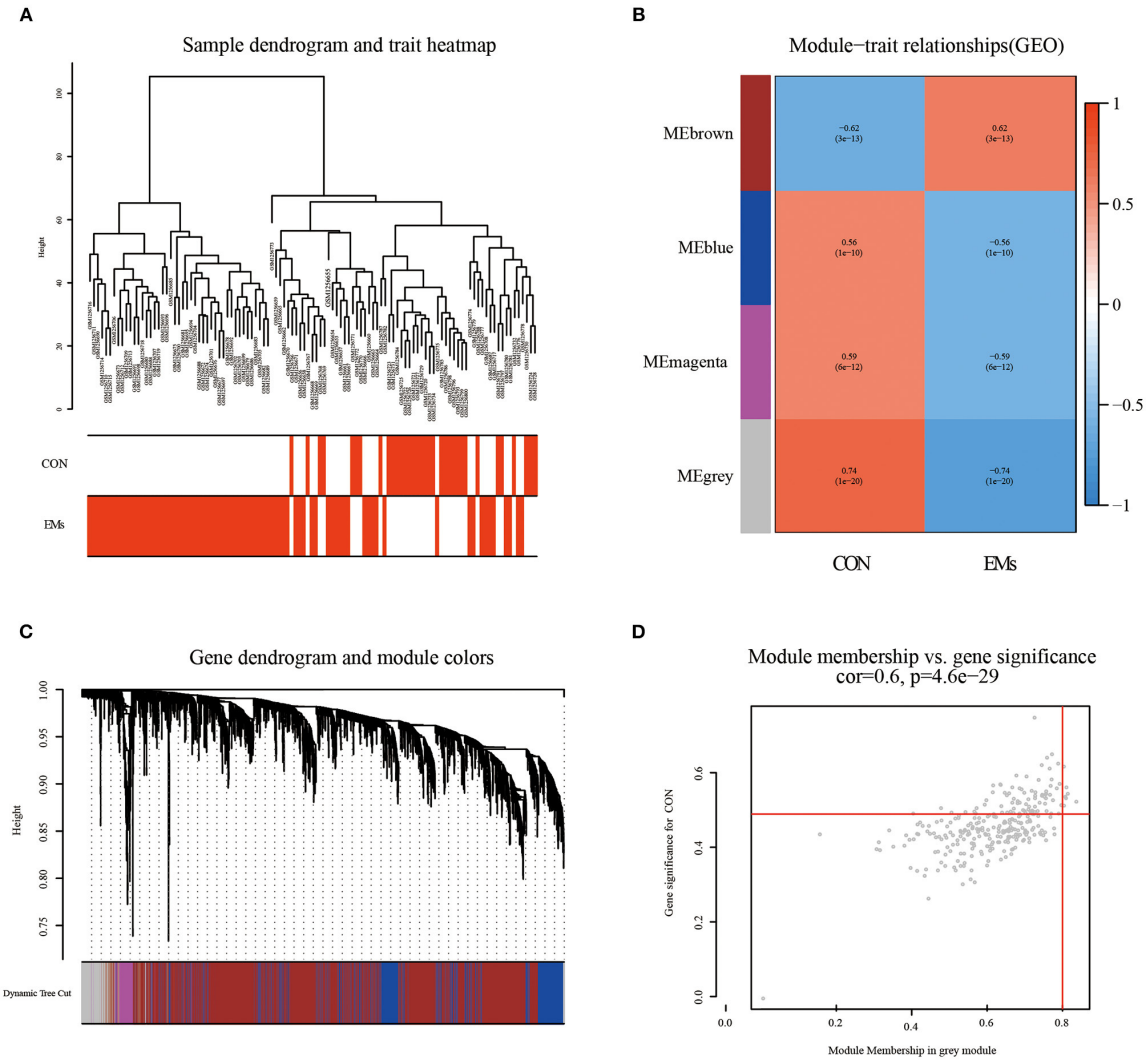
The results of WGCNA on the EMs dataset (GSE51981). **(A)** Dendrograms and corresponding feature heatmaps of the healthy and EMs samples. **(B)** Heatmap of correlations between gene modules and clinical features, with each grid showing the correlation between gene modules and clinical features. **(C)** Cluster dendrogram of co-expressed genes in EMs. **(D)** Correlation analysis results of gray gene modules and EMs.

**Figure 3:**
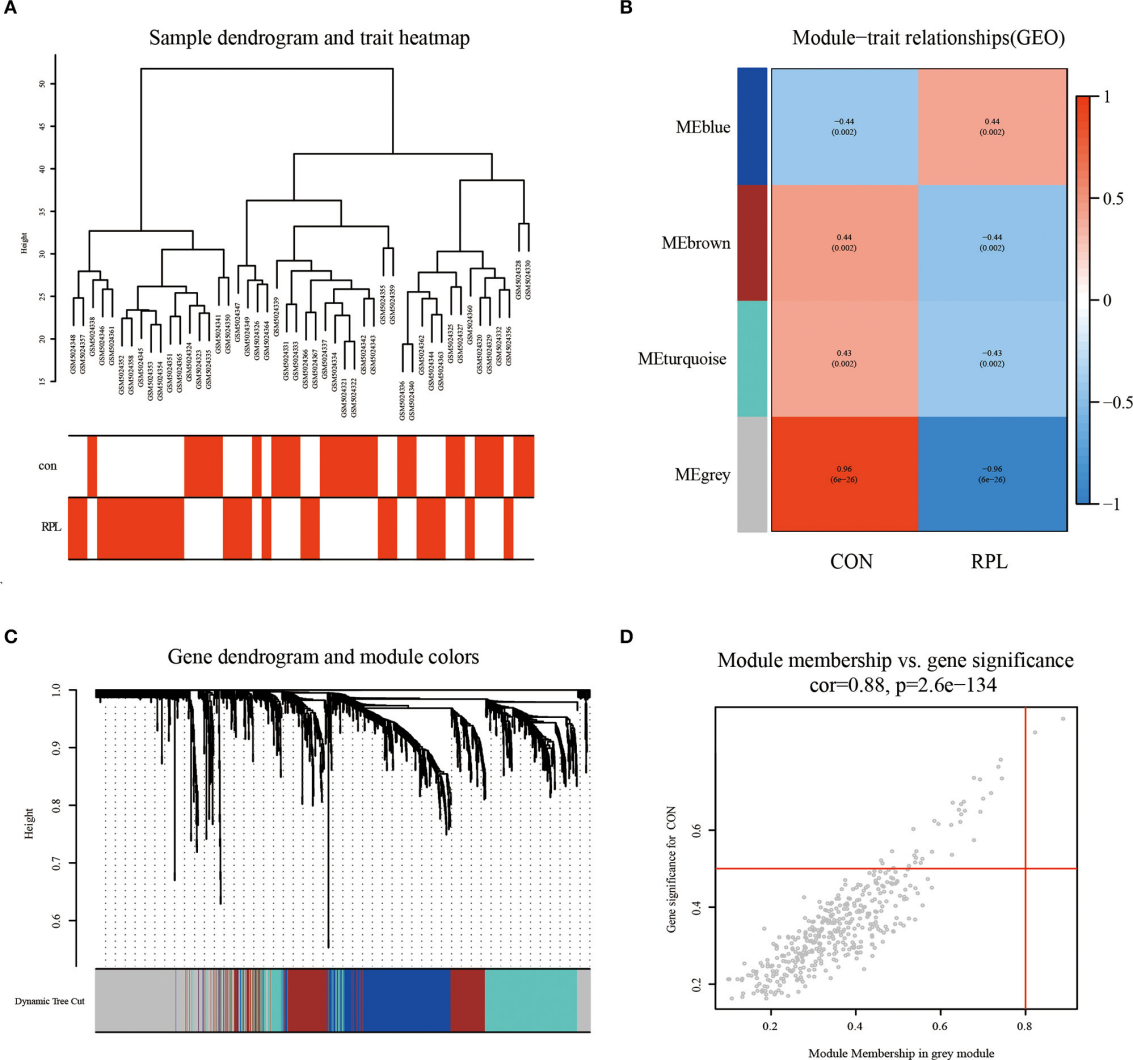
The results of WGCNA on the RPL dataset (GSE165004). **(A)** Dendrograms and corresponding feature heatmaps of the healthy and RPL samples. **(B)** Heatmap of correlations between gene modules and clinical features. **(C)** Cluster dendrogram of co-expressed genes in EMs. **(D)** Correlation analysis results of gray gene modules and EMs.

By clustering samples on the EMs dataset (GSE51981) and the RPL dataset (GSE165004), we found no abnormal samples. For these two datasets, Figures 2A, 3A showed sample clustering dendrograms and clinical feature heatmaps, respectively. We obtained 4 gene modules from the EMs dataset and the RPL dataset by clustering similar gene modules, respectively. Based on the correlation of gene modules with EMs and RPL, we created heatmaps of gene modules and the relationship between EMs and RPL (Figures 2B, 3B). The “brown,” “blue,” “magenta,” and “gray” gene modules had the high connection with EMs (brown gene module: *r* = 0.62, *p* = 3e-13; blue gene module: *r* = −0.56, *p* = 1e-10; magenta gene module: *r* = −0.59, *p* = 6e-12; gray gene module: *r* = −0.74, *p* = 1e-20). EMs was negatively linked with the blue, magenta, and gray gene modules, which contained 953, 150, and 283 genes (Supplementary Table 1), respectively. The brown gene module was positively correlated with EMs and contained 2,791 genes (Supplementary Table 1). Similarly, the gene modules “brown,” “turquoise,” and “gray,” which contain 293, 370, and 411 genes (Supplementary Table 2), were negatively connected with RPL (brown gene module: *r* = −0.44, *p* = 0.002; turquoise gene module: *r* = −0.43, *p* = 0.002; gray gene modules: *r* = −0.96, *p* = 6e-26). The “blue” gene module, which included 347 genes (Supplementary Table 2), was positively linked with RPL (blue gene module: *r* = 0.44, *p* = 0.002). Gene clustering dendrograms based on the top 5,000 differential genes were shown in Figures 2C, 3C. We identified the gene modules most linked with EMs and RPL by examining the correlation between gene modules and clinical characteristics (Figures 2D, 3D).

### Shared Genes of EMs and RPL

**Figure 4:**
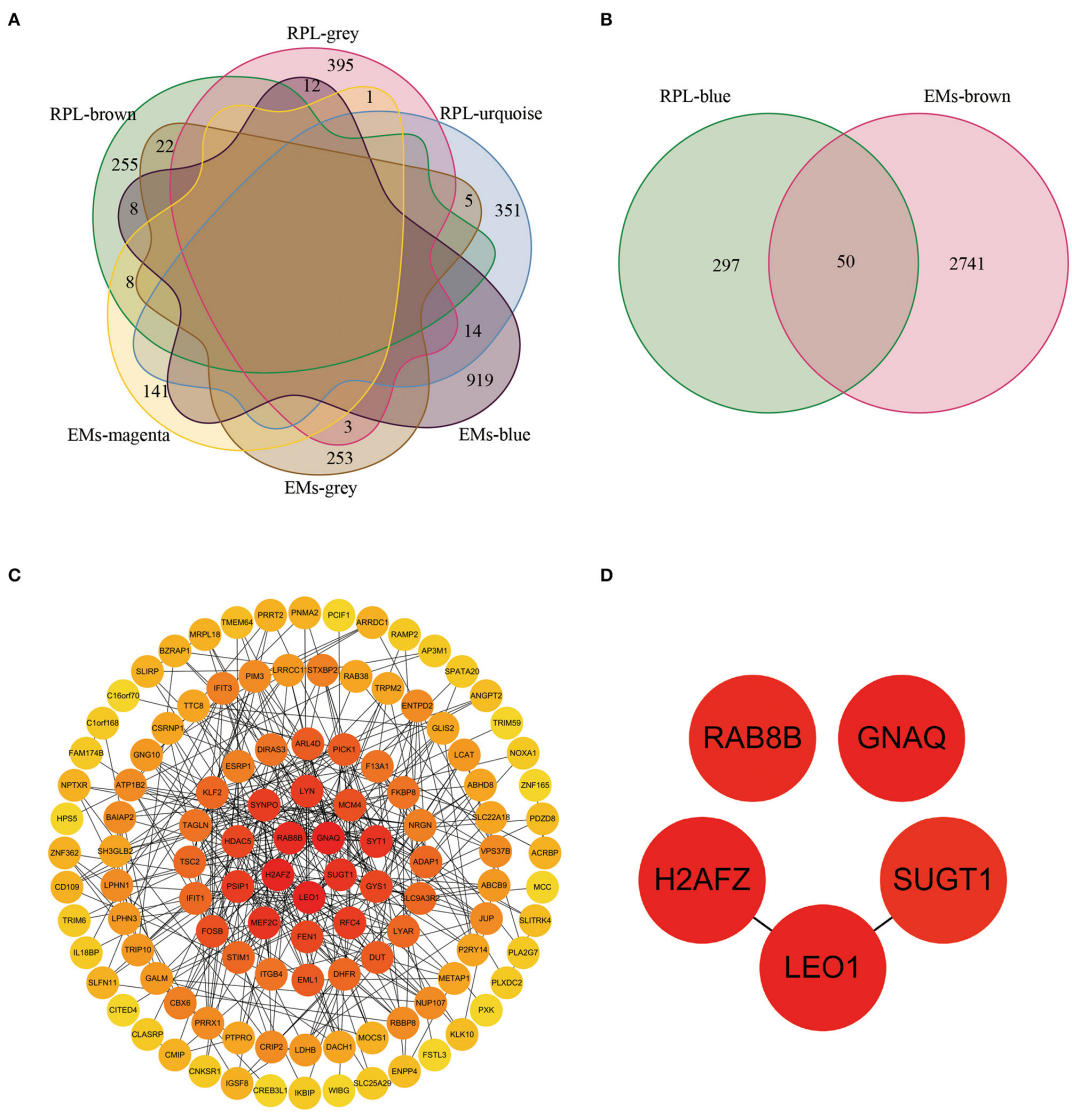
The shared genes and PPI network between the EMs-related gene modules and the RPL-related gene modules. **(A)** Shared genes between gene modules negatively correlated with EMs and gene modules negatively correlated with RPL (module correlation greater than 0.4). **(B)** Shared genes between gene modules positively correlated with EMs and gene modules positively correlated with RPL (module correlation greater than 0.4). **(C)** PPI network constructed by EMs and RPL shared genes. **(D)** Core shared genes for EMs and RPLs.

We obtained 73 and 50 genes (Figures 4A,B, Supplementary Table 3) by intersecting gene modules positively and negatively correlated with EM and RPL (gene module with disease correlation > 0.4), respectively. These 123 genes were identified as shared genes since they were closely associated with EMs and RPL.

### Construction of PPI Network

From the STRING database, we obtained the PPI network with 114 nodes and 738 edges (Supplementary Table 4). To further find the core genes of the PPI network, we imported the obtained PPI network into the Cytoscape software. Then, the degree value of each node in the PPI network was calculated through the CytoHubba plug-in. Figure 4C showed the PPI network containing node degree information. The node color was positively related to the degree value. The darker the color, the higher the degree value. RAB8B, GNAQ, H2AFZ, SUGT1, and LEO1 were the 5 nodes with the highest degree value (Figure 4D), which might be closely related to the occurrence, development, treatment and prognosis of EMs and RPL.

### Annotation of Shared Genes

**Figure 5:**
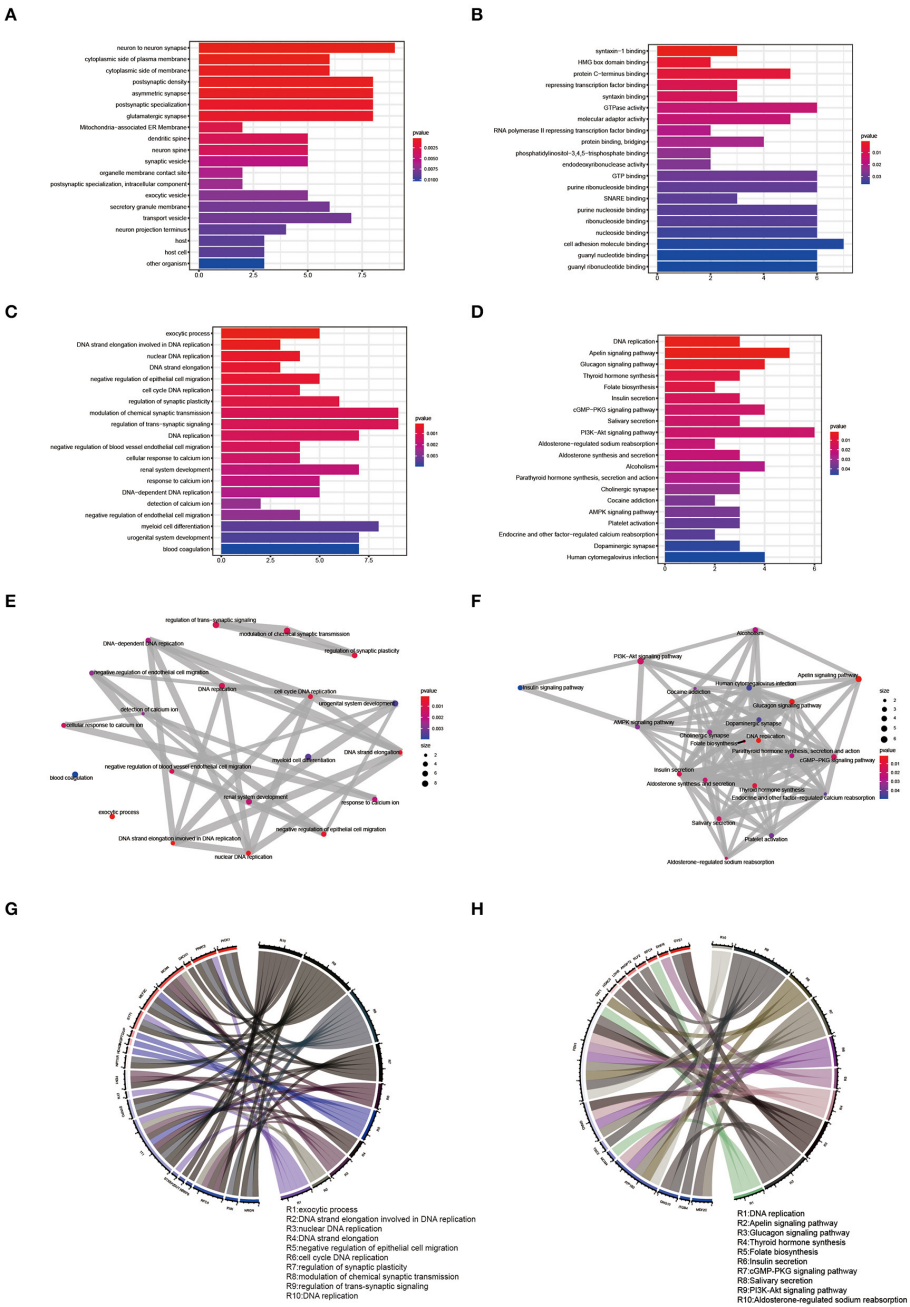
GO term and KEGG pathway enrichment analysis of shared genes. **(A–C)** Results of CC, MF, and BP of the GO term enrichment analysis of shared genes, respectively. **(D)** Results of KEGG pathway enrichment analysis of shared genes. **(E,F)** Correlation analysis of the GO BP term and KEGG pathway enrichment analysis results of shared genes. **(G,H)** Top ten GO BP term enrichment analysis results and the top ten KEGG pathway enrichment analysis results and their corresponding shared genes.

The R language “Venn” package was used to perform GO term and KEGG pathway enrichment analysis on 123 shared genes (Figures 5A–D, Supplementary Tables 5, 6). GO biological process analysis found that the top 20 analysis results included 6 DNA-related results, namely DNA strand elongation involved in DNA replication, nuclear DNA replication, DNA strand elongation, cell cycle DNA replication, and DNA replication. KEGG pathway enrichment analysis showed that DNA replication, Apelin signaling pathway, Glucagon signaling pathway, cGMP-PKG signaling pathway, PI3K-Akt signaling pathway, AMPK signaling pathway, Alcoholism, and Cocaine addiction might be involved in the pathological process of EMs and RPL. We used the R language “enrichplot” package to perform correlation analysis on the results to further investigate the link between these enrichment results of EMs and RPL (Figures 5E,F). We exhibited the top ten results of GO biological process enrichment analysis and KEGG pathway enrichment analysis with their corresponding genes in Figures 5G,H.

### Shared miRNAs of EMs and RPL

**Figure 6:**
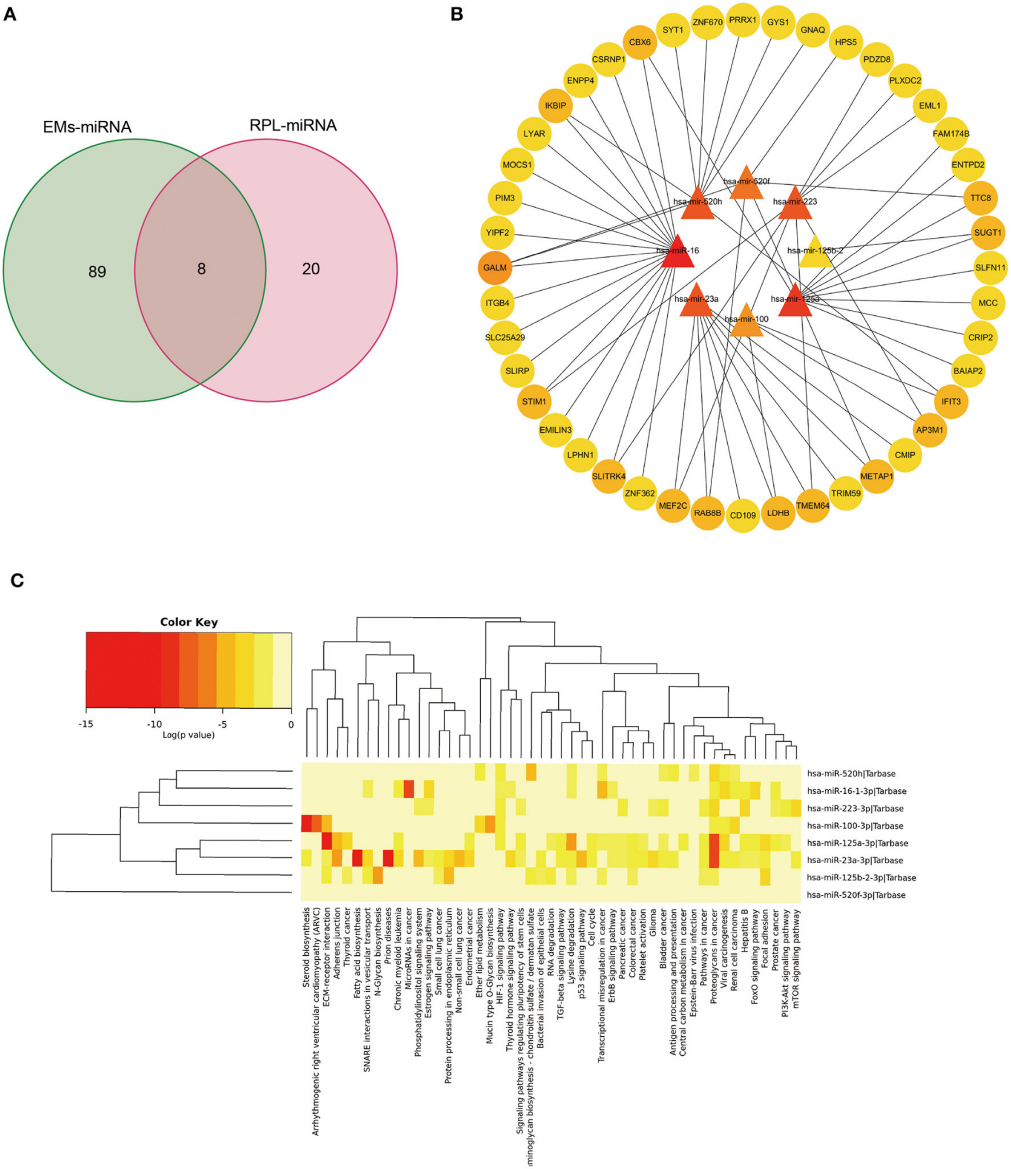
Bioinformatics analysis of genes and miRNAs shared by EMs and RPL. **(A)** Venn diagram of the intersection of EMs-related and RPL-related miRNAs. **(B)** Regulatory network of shared miRNAs and corresponding shared genes for EMs and RPL. **(C)** KEGG pathway enrichment analysis of shared miRNAs of EMs and RPL.

Through the HMDD database, we searched with EMs and abortion as keywords, respectively, and obtained 150 and 28 related miRNAs (Supplementary Table 7). After taking the intersection (Figure 6A), a total of 8 miRNAs were retrieved, including hsa-mir-100, hsa-mir-125a, hsa-mir-125b-2, hsa-mir-16, and hsa-mir-223, which were classified as shared miRNAs. We found target genes for 8 common miRNAs by searching the miRtarbase database (Supplementary Table 8). To establish the miRNAs-mRNAs regulation interaction related to EMs and RPL, the target genes of each shared miRNAs were intersected with the shared genes. Cytoscape software was used to visualize the regulatory connection between miRNAs and mRNAs (Figure 6B, Supplementary Table 9). MiRNAs were shown by triangles, while mRNAs were represented by circles. The higher the degree value, the darker the color. By using the mirPath v3.0 tool, we performed KEGG pathway enrichment analysis on 8 common miRNAs (Figure 6C, Supplementary Table 10). There were two overlapping pathways in the KEGG enrichment results of shared miRNAs and shared genes, namely the PI3K-Akt signaling pathway and platelet activation.

## Discussion

EMs is a chronic estrogen-dependent condition that can produce symptoms like dysmenorrhea, pelvic discomfort, and infertility, all of which have a negative impact on patients' physical and emotional health (10). EMs affects roughly 10% of women globally, yet the majority of them do not receive meaningful relief from the symptoms produced by EMs (11). EMs is the leading cause of infertility, with about two-fifths of infertile women suffering from EMs (12). Although hormone therapy and surgical therapy have been used to help EMs patients with infertility, the results are always disappointing (13). It is necessary to develop innovative and effective medical treatments. EMs, chronic endometritis, and other diseases that produce persistent endometrial inflammation disrupt the function of endometrium function, leading to early miscarriage and the development of RPL (9). However, the specific molecular process by which EMs leads to RPL is unknown. Bioinformatics is now widely used in the life sciences by integrating biology, computing, information engineering, mathematics and other disciplines to analyze massive amounts of data.

WGCNA is often used to find potential biomarkers and therapeutic targets by clustering highly related genes and performing correlation analysis between the clustered gene modules and clinical characteristics (14). In this study, we searched gene modules related to EMs and RPL by WGCNA, and obtained 123 shared genes. Among them, RAB8B, GNAQ, H2AFZ, SUGT1, and LEO1 may serve as potential therapeutic targets for patients with EM-induced RPL. As one of the most abundant protein families in the human body, the Rab family participates in the regulation of cell functions and is closely related to the transport of various components within cells (15). Multiple members of the Rab family can participate in different stages of autophagy, and RAB8B is mainly involved in regulating autophagosome maturation (16). RAB8B regulates the Wnt/β-catenin signaling pathway by affecting the activity and function of LRP6 (17). GNAQ, a member of the alpha subunit of the G protein, is mutated in approximately 80% of melanoma patients (18). By influencing the MAPK signaling pathway and the epithelial-mesenchymal transition, GNAQ enhances lung cancer cell proliferation and invasion (19). However, GNAQ activates the antioxidant properties of the Nrf2 protein and prevents cell damage (20). Placental oxidative stress has been linked to the development of RPL (21). The histone variant encoded by H2AFZ is an essential component of the nucleosome and affects the structure and function of eukaryotic chromatin (22). A recent prospective clinical study detected oocytes from EMs patients and healthy individuals by single-cell sequencing technology and found that H2AFZ was highly expressed in EMs patients' oocytes (23). As a co-chaperone of multiple proteins, SUGT1 participates in multiple cellular biological processes (24). The ability of SUGT1 to inhibit the proliferation of gastric cancer cells by regulating AKT phosphorylation makes it a potential therapeutic target for gastric cancer (25). The RNA polymerase-associated factor 1 complex contains LEO1, which is involved in chromatin remodeling and transcriptional elongation (26). LEO1 may be critical in zebrafish heart differentiation and neural crest cell population development (27). In summary, these 5 core genes are highly diverse in function and they may be involved in various stages of EM-induced RPL.

We used GO term enrichment analysis on shared genes to learn more about the molecular mechanisms of EMs-induced RPL. DNA replication appears to be implicated in the process of EMs-induced RPL, according to the results. Cell division happens constantly in the human body and is involved in different processes of human growth, development, and reproduction. Before cell division, various cellular components, including DNA replication, are frequently replicated (28). Several investigations have discovered that several epigenetic changes, including DNA methylation, are involved in the incidence and development of RPL at various stages through controlling the expression of critical genes in cell biological processes. Among them, DNA methylation-induced gene expression disorders and immunological imbalances affect all phases of embryonic development, which may be linked to RPL pathobiology (29). In addition, clinical studies have found that DNA damage induced by heavy metals may be related to the occurrence and development of RPL (30). Multiple modification mechanisms of epigenetics may be involved in the pathophysiology of EMs (31). Clinical studies have found that DNA methylation regulates genes related to endometrial function, which in turn affects the occurrence of many biological processes including cell proliferation and steroid hormone response (32). Furthermore, circulating cell-free nuclear DNA contained in human plasma and serum has been shown to be the biomarker for EMs (33).

We found 22 and 49 signaling pathways by using KEGG pathway enrichment analysis on shared genes and shared miRNAs, respectively. Two signaling pathways associated with EMs and RPL were discovered by intersecting the two analytical results, namely the PI3K-Akt signaling pathway and Platelet activation. By receiving signals from outside the cell, the PI3K-Akt signaling pathway is primarily engaged in the control of numerous cellular activities in the human body. The up-regulation of AXL and SHC1 could be related to the participation of the PI3K-Akt signaling pathway in the pathogenesis of EMs (34). As the pivotal pathway is closely associated with the progression of EMs, the PI3K/AKT signaling pathway is strongly linked to the regulation of functions such as proliferation, epithelial-mesenchymal transition, and invasion of ectopic endometrial stromal cells (35). The development of new drugs based on the PI3K/AKT signalling pathway for the treatment of EMs has great potential. Through the downstream matrix metalloproteinase 2, the PI3K/AKT signaling pathway is implicated in the migration of female trophoblast cells, which is linked to the incidence of RPL (36). PI3K/AKT signaling pathway is closely related to macrophage polarization. At the maternal-fetal interface, decidual macrophages govern immunological responses, and aberrant macrophage polarization is frequently linked to poor pregnancy timing, including RPL (37). Platelets are anucleated cells rich in many types of organelles. Through the regulation of the PI3K/AKT signaling pathway, platelets reduce the immune response of the microenvironment at the lesion site of EMs patients and then slow the progression of EMs (38). Platelets agglomerate in the lesions of patients with EMs, according to clinical investigations, may play a role in the advancement of EMs by causing microangiogenesis (39). Anti-platelet therapy can block epithelial-mesenchymal transition and fibrosis in EMs mice (40). Patients with RPL had the higher sensitivity to arachidonic acid, which promotes platelet aggregation (41). Platelet factor 4 and other blood biomarkers may be used to identify women at risk for RPL (42). In conclusion, the PI3K/AKT signaling pathway and platelet activation were both involved in the pathological process of EMs and RPL, so we speculated that these two pathways might be the potential mechanisms of EMs-induced RPL.

## Conclusion

In this study, the transcriptome sequencing data of EMs and RPL were analyzed by bioinformatics methods. For the first time, we investigated the underlying mechanisms (PI3K-Akt signaling pathway and platelet activation) and potential therapeutic targets (RAB8B, GNAQ, H2AFZ, SUGT1, and LEO1) of EMs-induced RPL by using multiple bioinformatics approaches, including WGCNA, to aid basic research, clinical diagnosis, and treatment of this disease.

## Data Availability Statement

The original contributions presented in the study are included in the article/Supplementary Material, further inquiries can be directed to the corresponding author/s.

## Author Contributions

HW, XL, WL, and CY developed the concept of the project. ZY collected and analyzed the data with the help of QM, JH, WZ, and HZ. HW, XL, WL, CY, and ZY wrote the manuscript. All authors have read and approved the final manuscript, reviewed and discussed the results, and contributed to the paper preparation.

## Funding

This study was supported by Shenzhen Key Laboratory Foundation (ZDSYS20200811143757022), Shenzhen Science and Technology Innovation Commission Project (Grant No. JCYJ20180302174235893), and Research funding for post-doctoral research in Shenzhen.

## Conflict of Interest

The authors declare that the research was conducted in the absence of any commercial or financial relationships that could be construed as a potential conflict of interest.

## Publisher's Note

All claims expressed in this article are solely those of the authors and do not necessarily represent those of their affiliated organizations, or those of the publisher, the editors and the reviewers. Any product that may be evaluated in this article, or claim that may be made by its manufacturer, is not guaranteed or endorsed by the publisher.

## Supplementary Material

Supplementary Table 1 Four gene modules associated with EMs.

Supplementary Table 2 Four gene modules associated with RPL.

Supplementary Table 3 Shared genes for EMs and RPL.

Supplementary Table 4 PPI network analysis results of shared genes.

Supplementary Table 5 GO term enrichment analysis results of shared genes.

Supplementary Table 6 KEGG pathway enrichment analysis results of shared genes.

Supplementary Table 7 MiRNAs associated with EMs and RPL.

Supplementary Table 8 Target genes corresponding to shared miRNAs.

Supplementary Table 9 MiRNAs-mRNAs regulatory network.

Supplementary Table 10 KEGG pathway enrichment analysis results of shared miRNAs.

## Abbreviations

EMs: endometriosis
RPL: recurrent pregnancy loss
GEO: gene expression omnibus
WGCNA: weighted gene co-expression network analysis
miRNA: microRNA
HMDD: human microRNA disease database
TOM: topological overlap matrix
PPI: protein-protein interaction
GO: gene ontology
KEGG: kyoto encyclopedia of genes and genomes.
